# Socioeconomic and Demographic Factors for Spousal Resemblance in Obesity Status and Habitual Physical Activity in the United States

**DOI:** 10.1155/2014/703215

**Published:** 2014-09-23

**Authors:** Hsin-Jen Chen, Yinghui Liu, Youfa Wang

**Affiliations:** ^1^Department of International Health Human Nutrition Program, Bloomberg School of Public Health, Johns Hopkins University, Baltimore, MD 21205, USA; ^2^Institute of Public Health, National Yang-Ming University, Taipei 11219, Taiwan; ^3^Department of Epidemiology and Environmental Health, School of Public Health and Health Professions and School of Medicine and Biomedical Sciences, University at Buffalo, State University of New York, Farber Hall, Room 270, 3435 Main Street, Buffalo, NY 14214-8001, USA

## Abstract

Studies suggested that the married population has an increased risk of obesity and assimilation between spouses' body weight. We examined what factors may affect married spouses' resemblance in weight status and habitual physical activity (HPA) and the association of obesity/HPA with spouses' sociodemoeconomic characteristics and lifestyles. Medical Expenditure Panel Survey data of 11,403 adult married couples in the US during years 2006–2008 were used. Absolute-scale difference and relative-scale resemblance indices (correlation and kappa coefficients) in body mass index (BMI) and HPA were estimated by couples' socioeconomic and demographic characteristics. We found that spousal difference in BMI was smaller for couples with a lower household income, for who were both unemployed, and for older spouses. Correlation coefficient between spouses' BMI was 0.24, differing by race/ethnicity and family size. Kappa coefficient for weight status (obesity: BMI ≥ 30, overweight: 30 > BMI ≥ 25) was 0.11 and 0.35 for HPA. Never-working women's husbands had lower odds of obesity than employed women's husbands (OR = 0.69 (95% CI = 0.53–0.89)). Men's unemployment status was associated with wives' greater odds of obesity (OR = 1.31 (95% CI = 1.01–1.71)). HPA was associated with men's employment status and income level, but not with women's. The population representative survey showed that spousal resemblance in weight status and HPA varied with socioeconomic and demographic factors.

## 1. Introduction

Married individuals have better health and healthier behaviors than the unmarried, including a lower prevalence of smoking, better self-assessed health [1], lower risks of cancer [2], and lower mortality [3, 4]. However, married people are more likely to be overweight or obese [1, 5, 6] and tend to have decreased physical activity/fitness and increased body mass index (BMI) [7–11]. This paradox may be related to the convergence of lifestyle and behaviors between spouses after marriage. As more than two-thirds of the US adults are overweight or obese [12], understanding spousal resemblances, the interspousal influence on obesity, and its related behaviors could provide insight into the family processes of obesity for the married couples.

Spousal resemblance in weight status has been reported [13, 14]: a meta-analysis concluded the correlation coefficient in BMI between spouses which was about 0.15 (95% CI = 0.05–0.25) [13]. The Framingham Study showed that spouse's obesity and siblings' obesity had similar odds ratio for ones' chance of becoming obese [15]. As spouses share fewer common genes than siblings do, spousal resemblance in weight status results from shared environments, common lifestyles rooted in the same cultural background, and assortative selection of marriage [16]. Similar weight-related behaviors between spouses, such as dietary behaviors, physical activity, and tobacco smoking, were reported [16–18]. Both healthy and unhealthy behaviors can diffuse and exchange between the partners, while the family condition and context could affect the passages of obesity-related behaviors and lifestyle between wife and husband [11, 19, 20].

Despite the clear evidence on spousal resemblance in lifestyle and weight status, little is known about how family contextual factors, such as household socioeconomic and couples' demographic characteristics, could influence the degree of spousal BMI resemblance. For instance, socioeconomic status (SES) is associated with obesity [12]. Spouses under the same family socioeconomic condition share resources and stresses in life, which in turn contribute to the spouses' obesity-related behaviors such as eating and health status. Identifying the family contextual factors associated with the spousal resemblance would help direct attention on families/spouses needing assistance.

Using recent US nationally representative data, we examined the absolute- and relative-scale spousal resemblance in weight status and habitual physical activity (HPA) and explored how the resemblance varied with family- and couple-level characteristics. In addition, for further understanding the potential influence between spouses, we studied how people's weight status and HPA were related to their own and the spouses' socioeconomic and demographic characteristics.

## 2. Methods

### 2.1. The Medical Expenditure Panel Survey (MEPS)

The MEPS is an ongoing nationally representative survey of the civilian noninstitutionalized population, conducted annually since 1996, using an overlapping panel design. The sampling frame is the participating households in the previous year's National Health Interview Survey, based on a complex sampling structure designed to produce nationally representative estimates [21]. During the interviews of the MEPS, household respondent reported demographic characteristics, health conditions, health status including BMI and level of physical activity, health insurance coverage, and employment for all household members. The full year-consolidated data files from 2006 to 2008 were used in this study. Data were analyzed in 2011-2012.

### 2.2. Study Population

Subjects without a specific spouse identifier were ineligible for this study. Subjects with medical conditions during the survey year that would considerably affect body weight were excluded, such as pregnancy, HIV/AIDS, cancer, diabetes, and thyroid diseases. These conditions were identified based on the MEPS Medical Condition Files. Because of the small sample size, 13 pairs of spouses aged <18 and 10 pairs of same-sex couples were excluded. We also excluded married but separated couples and couples that had discrepant reports on marital status. Spouses having missing or unreasonable BMI values (<13 and >52, the 1.5 interquartile ranges (IQRs) lower than the bottom BMI quartile and 3 IQRs higher than the top BMI quartile) were excluded from final analysis. The final sample size was 22,806 married subjects, that is, 11,403 couples of adult, opposite-sex, cohabiting, and relatively healthy spouses. As sampling weight is required for nationally representative statistics, eligible data of nonzero sampling weight from 10,843 men and 10,937 women were included for individual-level analysis.

For couple-level statistics, we changed the original person-level data into couple-level structure. Considering nonzero family-level sampling weights, 11,254 out of the 11,403 couples were eligible for analysis.

### 2.3. Key Study Variables

#### 2.3.1. Outcome Variables

*(1) Weight Status.* BMI (kg/m^2^) was calculated from subjects' body weight and height reported by household respondents. Overweight and obesity were defined using BMI cut points of 25.0–29.9 and ≥30.0, respectively.

*(2) Habitual Physical Activity (HPA).* HPA was a dichotomous outcome, reported by the household respondent to the following question, “Do you now spend half an hour or more in moderate or vigorous physical activity at least three times a week?”

#### 2.3.2. Individual-Level Factors

These included demographics (age, race/ethnicity, marital status, rural/urban residence, family size, and birth origin) and SES characteristics (education years, income, and employment status) and current smoking status (yes/no).

Races/ethnicities were categorized into non-Hispanic (NH) white, NH black, Hispanic, and other. Individuals' annual income was categorized into <US$10,000, US$10,000–24,999, US$25,000–47,999, and US$48,000+. Employment statuses included employed, retired, never worked, and unemployed. All covariates included category for missing values. Indicators for survey years and for the subject's responding to the household questionnaires were included to control for survey procedure-related factors.

#### 2.3.3. Household- and Couple-Level Variables

For couple-level analysis, household-level variables included household income category (poor/near poor: <1.25 poverty line; low income: 1.25–1.9 poverty line; middle income: 2.0–3.9 poverty line; high income: ≥4 poverty line, as derived from the poverty statistics by the Current Population Survey), receiving food stamps in the past year (yes versus no), and family size (2, 3-4, or ≥5 persons). The poverty line was adjusted for family size and composition, a dollar value threshold to determine who was in poverty [22]. Couple-level variables were created by the combination of spouses' individual characteristics. Mean age of a couple was categorized as <30, 30-39, 40–54, and ≥55 years old. The combination of the couple's unemployment status included “both unemployed,” “only husband unemployed,” “only wife unemployed,” and “both employed.”

The race/ethnicity combinations of spouses were “both NH white,” “both NH black,” “both Hispanic,” “both were other race/ethnicity,” and “interracial/ethnic.” Couples' HPA statuses were combined into both, only husband, only wife, and neither of spouses having HPA.

### 2.4. Statistical Analysis

We did individual- and couple-level analyses and used the complex sampling design for estimating representative statistics and appropriate variance. Using the survey procedures of SAS 9.2, we applied person-level sample weights for the individual-level analyses and family-level sample weights for the couple-level analyses.

Couple-level analysis assessed spousal resemblance at two dimensions: relative-scale association and absolute-scale difference. Previous studies about spousal resemblance in weight status mainly focused on the degree of association or concordance between husbands' and wives' health outcomes and behaviors [13, 16, 23]. Nevertheless, the relative-scale dyadic association and concordance statistics for weight resemblance only indicate the tendency to convergence of weight status and obesity-related behaviors [24, 25], but the absolute similarity between spouses is not shown by these statistics. In the present study, we used both relative- and absolute-scale statistics of resemblance. The relative association between spouses' weights and HPA was measured by correlation and kappa coefficients and odds ratios (ORs), while the absolute similarity was measured by the direct differences of the outcomes between husbands and wives (see below). For the latter, the larger the spousal difference, the smaller their similarity. Linear regression examined the husband-wife difference in BMI across household-/couple-level characteristics.

Pearson's correlation coefficients between spouses' BMI were estimated by regressing the standard scores of the husbands' BMI on the standard scores of the wives' BMI. Regression coefficient of the husbands' and the wives' standard scores is rescaled correlation coefficient [26, 27]. The reasons that we took a modeling strategy to estimate the correlation coefficient in BMI were (1) to accommodate the complex survey sampling design in standard error calculation and (2) to adjust for covariates, including age, education years, race/ethnicity, HPA, current smoking status, individual income, employment status, survey year, indicator of respondent, and missingness indicators. Differences in correlation coefficients by household-/couple-level characteristic strata were tested by the interaction terms of wife's BMI standard score and the relevant strata.

As for categorical outcomes, kappa coefficients for weight status (normal weight, overweight, and obese) and HPA (dichotomous) between spouses were calculated to measure crude agreement [28]. Kappa coefficient is a nonparametric statistics; its variance was estimated using Fay's balanced repeated replication method, and Fay's coefficient was set to 0.5.

To examine the spousal association in weight status, household-/couple-level logistic regression models were fitted to estimate the odds ratio (OR) between husbands' (dependent) and wives' (independent) obesity/HPA, with adjustment for individual-level variables, that is, husband's and wife's ages, education years, races/ethnicities, HPA (when the outcome was weight status), current smoking statuses, individual incomes, and employment statuses.

Finally, individual-level logistic regression examined how one's own and spouse's characteristics were associated with one's own obesity/HPA. One's own and spouse's individual-level lifestyles and SES were included in model altogether to study the differential associations between spouses.

## 3. Results

Among married couples in the US, husbands were older, had greater BMI, and were more physically active than wives. Wives and husbands had similar distributions of race/ethnicity and education. Men earned more and had a higher employment rate than wives (Table 1).

The husband-wife differences in BMI varied with household-/couple-level characteristics (Table 2). Except for the NH black couples, wives had significantly lower BMI than husbands for all the other racial/ethnic couples (*P* < 0.05). Meanwhile, wives' lower BMI than husbands' was not significant for couples where only the husband was physically active, who were both unemployed, who had low income or poor/near poor status, or who received food stamp in the past year. Spousal difference in BMI was significantly associated with food stamp reception and the spouses' HPA.

The unadjusted correlation coefficient between wives' and husbands' BMI was 0.26. After adjustment for covariates, the correlation coefficients were 0.24. The adjusted correlation coefficient in BMI was the lowest among couples of different races/ethnicities (significantly lower than NH white couples, *P* = 0.014). Couples living by themselves had significantly lower spousal correlation in BMI than couples in households of ≥5 family members (*P* = 0.034). Compared to both-employed couples, the correlation was lower for those where only the husbands were unemployed (*P* = 0.005); compared to both-physically active couples, the correlation was lower for those where only the husbands were inactive (*P* = 0.025).

Kappa coefficients (range: 0.05–0.16) suggested a low resemblance in weight status between spouses, without adjustment for any covariates. Adjusted logistic models showed that one's odds to be obese were associated with the spouse's obesity status (OR = 2.52, 95% CI: 2.2–2.9; Table 2). The variation patterns in ORs for obesity across subgroups were similar to those variation patterns in kappa and adjusted correlation coefficients.

Men's and women's obesity were associated with different sets of their spouses' characteristics (Table 3). Controlling for the spouse's age, men's obesity status was inversely associated with their own age. Subject's obesity was only associated with their own HPA, not their spouse's. Also for men, current smokers had lower odds of being obese than nonsmokers, but wives' smoking status was associated with husbands' greater odds of obesity. Black men's wives were more likely to be obese than white men's wives, while men's obesity was not associated with wives' race/ethnicity.

As for SES, husbands' education was associated with lower odds of his own obesity status and his wife's obesity status. Women whose husbands earned <US$48,000/year had higher OR of obesity than those whose husbands earned ≥US$48,000/year. Never-working wives' husbands were less likely to be obese compared to working wives' husbands. On the other hand, wives were more likely to be obese if husbands were unemployed than if husbands were employed.

HPA was more common among husbands than among wives. The overall kappa coefficient for HPA between wives and husbands was 0.35. The variation patterns of kappa coefficients by strata were similar to the patterns of stratified ORs (Table 4).

Hispanic couples had the strongest concordance in HPA among the racial/ethnic groups. The proportion of having HPA was also the lowest for Hispanic couples. Using household income of 200% of the poverty line as a cut-off, the OR for spouse's HPA was significantly greater for the lower-income families than the OR for the richer families. However, low-income couples were less likely to engage in HPA than their richer counterparts. A similar pattern was found between couples ever-receiving food stamps and their counterparts.

Table 5 shows that one's own HPA was highly associated with the spouse's HPA. HPA was less prevalent among smokers than among nonsmokers. Wives' HPA was positively associated with husbands' higher education and income. Husbands' HPA was not significantly associated with wives' socioeconomic characteristics.

## 4. Discussion 

This study based on a national survey shed light on the family socioeconomic context for spousal resemblance in weight status and HPA in the US. As our results show, the spousal resemblance in weight status and HPA need to be considered in combination with the absolute levels of the outcomes for husbands and wives. As two dimensions of resemblance, relative-scale and absolute-scale indices do not necessarily coincide with each other. For example, the greater correlation coefficient in BMI for NH black spouses and the similarly high BMI among black husbands and wives suggest the black spouses' strong ties at high BMI. Similarly, poorer couples' stronger concordance in HPA with lower prevalence of HPA would draw attention to spouses with such socioeconomic condition.

Some studies suggested that the spousal resemblance attributable to assortative mating would decrease with marriage duration [13, 29, 30]. Similarly, we found a pattern that the spousal correlation in BMI attenuated with the couple's mean age, though husbands' and wives' mean BMI values became closer for older couples (not significant yet). Note that marriage duration data were not available in MEPS, while the couples' mean age could serve as a crude indicator of it. Although men's and women's BMI both increased after marriage [7, 10], BMI changing in the same direction may not necessarily mean an increasing concordance, if husband's and wife's BMI increments (change in “level”) are not parallel (change in “shape”). A weight-loss intervention study found that patients' spouses would have parallel changes in dietary behaviors and weight status [31]. Contrarily, the spouses of gastric bypass surgery patients in another study showed significant weight gain after the surgery [32]. Longitudinal resemblance study is needed to understand how familial and spousal characteristics may contribute to spouses' differential weight change.

Lower SES, such as lower income or unemployment, was associated with adults' lower HPA but was also associated with stronger spousal resemblance in HPA. These findings suggest that the spousal influence on habits might be weaker in families of higher socioeconomic status than in families of lower socioeconomic status. For example, as both spouses are full-time employed and subsequently have more family income, the different activity schedules between the spouses may reduce the resemblance of habits and lifestyle between spouses. On the other hand, the stronger spousal resemblance of habits in lower SES families is not necessary positive for public health, since unhealthy behaviors could be also easily shared between these spouses. As our data showed, spousal resemblance in weight status in families receiving food stamp was significantly greater than in families not receiving food stamp. In other words, spouses of higher SES and lower SES families face different issues about lifestyle and healthy behavior dynamics.

Both obesity status and HPA were associated with ones' own and their spouses' SES and/or lifestyles. Men were less likely to be obese when their wives were nonemployed than employed. Maternal employment has been associated with childhood obesity, which was probably because of less time on food preparation and shared activities between mother and child [33, 34]. Our study also suggests a spillover effect of wives' employment on husbands' obesity, consistent with other studies in the US and France [35, 36]. On the contrary, husbands' unemployment status and lower income were associated with wives' greater odds of obesity and lower odds of HPA.

The opposite association of husbands' and wives' employment status on spouse's obesity could result from the current gender norms of household labor division [37]. The American Time Use Survey in 2010 showed that, among married adults having children, unemployed and employed husbands on average spent 1.87 and 1.29 hours/day on housework, respectively, while unemployed and employed wives spent 2.91 and 2.14 hours/day [38]. On the other hand, household finances might be impacted more by husband's rather than wife's being unemployed in a society where there is a gender wage gap. While lower income is associated with more consumption of energy-dense foods [39], how the current gender roles in the division of labor in US families contribute to obesity is an underexplored topic.

To the best of our knowledge, this is the first comprehensive and vigorous examination of spousal resemblance in weight status and HPA. The present study has several strengths. First, it is based on US nationally representative data from a large sample. The results can be generalized to the married adult population in the US. Second, we studied the spousal resemblance from different perspectives. Similarity at absolute scale was considered to help identify the subgroups trapped in the environment at higher health risk, in complement to the strength of concordance reflected by the relative-scale indices. Third, we looked at the couple-level resemblance in weight status and conducted individual-level analysis to examine the differential association between spouses' characteristics and their obesity/HPA.

A main limitation of this study is the cross-sectional design, which shows the distributions and patterns of associations but cannot confirmatively make causal inferences. Second, BMI was calculated based on self- or proxy-reported weight and height. Heavier adults tend to underreport their body weight and underweight people overreport body weight [40]. The different resemblance statistics were applied to examine the sensitivity of results under reporting error in the distribution. Pearson's correlation coefficient summarizes the association through the full range of distribution, although the tail distributions may leverage its estimate. Kappa coefficient can be less sensitive to the tail distribution, but categorization can lose the richer information that continuous measurements can give. This suggests the value of examining resemblance using multiple statistics [41]. The consistent patterns of relative resemblance indices across subgroups suggest that the potential impact of report bias of BMI could be limited. Third, MEPS does not collect detailed information about marriage, for example, marriage duration and number of marriages in life. Nevertheless, the current marriage status provides a snapshot of the married population in the US.

## 5. Conclusion

The US nationally representative data shows a modest but clear spousal resemblance in physical activity and weight status. Although marriage protects health in general, it may also invite lower fitness and obesity, especially in disadvantaged populations. Spouses in households receiving food stamps, with larger family sizes and with lower incomes, had a stronger resemblance in weight status/HPA and had higher BMI level or were less active. The stronger spousal resemblance in HPA and/or weight status in lower socioeconomic families should be of more attention, and social welfare program such as the federal funded food assistance program should provide additional education to reduce spouses' shared unhealthy habits and lifestyles. These suggest that the households with these characteristics would have stronger endogenous forces driving household members toward less satisfactory health behaviors and weight status.

## Figures and Tables

**Table 1:** Characteristics of husbands and wives in the US (MEPS 2006–2008).

	Husbands (*n* = 10843)		Wives (*n* = 10937)	
	Mean of %	95% CI	Mean of %	95% CI
Age (years)	46.0	(45.6, 46.4)	43.5	(43.1, 44.0)
BMI (kg/m^2^)	27.9	(27.8, 28.1)	26.4	(26.2, 26.5)
BMI category (%)				
<25	25.6	(24.4, 26.8)	48.0	(46.6, 49.5)
25–29	46.9	(45.7, 48.1)	29.1	(28.0, 30.2)
≥30	27.5	(26.3, 28.7)	22.9	(21.7, 24.1)
Habitual physical activity (%)^1^	61.7	(60.2, 63.1)	58.2	(56.9, 59.6)
Current smokers (%)	19.7	(18.5, 20.9)	14.8	(13.7, 15.9)
Education attainment (%)				
<12 years	13.6	(12.6, 14.6)	11.8	(10.8, 12.7)
12 years	29.7	(28.2, 31.1)	28.8	(27.5, 30.0)
13–15 years	21.3	(20.1, 22.6)	24.2	(22.9, 25.5)
≥16 years	35.4	(33.8, 37.0)	35.2	(33.6, 36.9)
Race/ethnicity (%)				
NH white	71.5	(69.7, 73.2)	71.3	(69.6, 73.0)
NH black	7.6	(6.8, 8.3)	7.2	(6.5, 8.0)
Hispanic	14.2	(12.7, 15.7)	14.1	(12.7, 15.6)
Other	6.8	(5.9, 7.7)	7.3	(6.3, 8.2)
Individual annual income category (%)				
<US$10,000	8.1	(7.5, 8.8)	26.4	(25.3, 27.6)
US$10,000–24,999	20.6	(19.6, 21.7)	23.5	(22.5, 24.5)
US$25,000–47,999	32.3	(31.1, 33.4)	26.6	(25.6, 27.7)
≥US$48,000	38.9	(37.5, 40.4)	23.4	(22.1, 24.7)
Employment status (%)				
Never work	0.8	(0.6, 1.0)	8.9	(7.9, 9.8)
Retired	6.7	(6.1, 7.4)	4.3	(3.7, 4.9)
Unemployed	4.8	(4.4, 5.3)	16.3	(15.3, 17.3)
Employed	87.6	(86.8, 88.4)	70.5	(69.2, 71.7)
Interview respondent (%)	27.6	(26.4, 28.8)	72.7	(71.5, 73.8)

^1^Moderate to vigorous intensity physical activity ≥3 times/week.

**Table 2:** Relative association and absolute similarity in weight status among the US married spouses, stratified by household/couple-level characteristics (MEPS 2006–2008).

	*N* ^ $^ of spouses	Absolute				Relative associations							
		(1) Similarity				(2) Pearson's correlation coefficients			(3) Kappa coefficients between spouses weight status		(4) ORs between wife's and husband's obesity status		
		Mean BMI				BMI standard scores			Normal/overweight/obesity		Obesity		
		Husband	Wife	*D* ^£^		Partial *r* ^†^	95% CI		*k*	95% CI	OR^†^	95% CI	
Whole sample	11254 (100%)	27.9	26.3	**1.6 **		**0.24**	(0.21, 0.27)		**0.107**	(0.107, 0.108)	**2.5**	(2.2, 2.9)	
Stratified by													
Couple's race/ethnicity													
Both NH white^(ref)^	5758 (69%)	28.0	26.0	**1.9 **		**0.26**	(0.22, 0.30)		**0.102**	(0.101, 0.102)	**2.8**	(2.3, 3.4)	
Both NH black	1127 (7%)	29.0	29.2	**−0.2 **		**0.21**	(0.14, 0.28)		**0.059**	(0.053, 0.066)	**1.7**	(1.2,2.3)	∗
Both Hispanic	2646 (12%)	28.3	27.8	**0.4 **		**0.20**	(0.14, 0.26)		**0.099**	(0.096, 0.102)	**2.3**	(1.8, 2.8)	
Both were other	755 (5%)	25.4	23.5	**1.9 **		**0.29**	(0.18, 0.41)		**0.094**	(0.085, 0.104)	**3.4**	(1.3, 9.1)	
Interracial/ethnic	968 (7%)	28.0	26.1	**1.9 **		**0.14**	(0.06, 0.23)	∗	**0.089**	(0.079, 0.098)	**1.8**	(1.2, 2.8)	
Couple's mean age													
<30 y	1540 (13%)	27.6	25.6	**1.9 **		**0.27**	(0.19, 0.35)		**0.090**	(0.085, 0.095)	**3.0**	(2.1, 4.4)	
30–39 y	3227 (26%)	28.1	26.3	**1.8 **		**0.28**	(0.23, 0.34)		**0.116**	(0.114, 0.119)	**2.9**	(2.3, 3.7)	
40–54 y	4241 (39%)	28.2	26.5	**1.7 **		**0.21**	(0.16, 0.27)		**0.116**	(0.115, 0.118)	**2.4**	(1.9, 3.1)	
≥55 years	2246 (22%)	27.4	26.4	**1.0 **		**0.20**	(0.14, 0.27)		**0.081**	(0.078, 0.084)	**1.9**	(1.3, 2.8)	
Physical activity													
Both active	4629 (45%)	27.4	25.4	**2.0 **		**0.24**	(0.20, 0.28)		**0.102**	(0.101, 0.103)	**2.9**	(2.4, 3.5)	
Only husband active	2025 (18%)	27.6	27.4	**0.2 **	∗∗∗	**0.23**	(0.17, 0.29)		**0.113**	(0.108, 0.117)	**2.8**	(2.1, 3.8)	
Only wife active	1438 (14%)	28.5	25.8	**2.7 **	∗∗∗	**0.18**	(0.10, 0.25)	∗	**0.052**	(0.048, 0.056)	**2.0**	(1.4, 2.8)	
Both inactive^(ref)^	3092 (24%)	28.7	27.5	**1.2 **		**0.28**	(0.22, 0.33)		**0.128**	(0.126, 0.130)	**2.3**	(1.8, 2.8)	
Family size													
2 persons	3345 (34%)	27.5	25.8	**1.7 **		**0.20**	(0.14, 0.26)	∗	**0.089**	(0.087, 0.091)	**2.1**	(1.6, 2.8)	
3-4 persons	5162 (47%)	28.0	26.4	**1.6 **		**0.24**	(0.20, 0.28)		**0.109**	(0.107, 0.110)	**2.6**	(2.1, 3.1)	
≥5 persons^(ref)^	2747 (18%)	28.5	27.0	**1.4 **		**0.30**	(0.23, 0.36)		**0.130**	(0.127, 0.132)	**3.1**	(2.3, 4.0)	
Employment condition													
Both unemployed	873 (7%)	27.1	26.7	**0.4 **		**0.22**	(0.11, 0.32)		**0.075**	(0.068, 0.083)	**2.2**	(1.4, 3.6)	
Only husband unemployed	625 (5%)	28.0	26.9	**1.1 **		**0.09**	(−0.01, 0.20)	∗∗	**0.053**	(0.042, 0.063)	**1.3**	(0.7, 2.3)	∗∗
Only wife unemployed	2835 (22%)	27.7	26.3	**1.4 **		**0.23**	(0.18, 0.29)		**0.109**	(0.107, 0.112)	**2.4**	(1.8, 3.1)	
Both employed^(ref)^	6899 (65%)	28.1	26.2	**1.9 **		**0.26**	(0.22, 0.30)		**0.115**	(0.114, 0.116)	**2.8**	(2.3, 3.3)	
Household income level													
Poor/near poor (<1.25 poverty line)	1611 (8%)	28.0	27.6	**0.3 **		**0.25**	(0.18, 0.32)		**0.107**	(0.102, 0.112)	**2.3**	(1.8, 3.1)	
Low income (1.25–2.0 poverty line)	1564 (10%)	27.9	27.4	**0.5 **		**0.26**	(0.18, 0.33)		**0.092**	(0.087, 0.097)	**2.5**	(1.8, 3.4)	
Middle income (2.0–4.0 poverty line)	3545 (32%)	28.2	27.0	**1.2 **		**0.25**	(0.19, 0.30)		**0.117**	(0.115, 0.119)	**2.6**	(2.1, 3.2)	
High income (≥4.0 poverty line)	4534 (50%)	27.7	25.5	**2.3 **		**0.23**	(0.19, 0.27)		**0.102**	(0.101, 0.104)	**2.6**	(2.1, 3.2)	
Receiving food stamp last year													
Yes	705 (3%)	28.5	29.2	**−0.6 **	∗∗	**0.27**	(0.17, 0.37)		**0.162**	(0.153, 0.171)	**3.0**	(2.0, 4.5)	
No^(ref)^	10549 (97%)	27.9	26.2	**1.7 **		**0.24**	(0.21, 0.27)		**0.105**	(0.104, 0.105)	**2.5**	(2.2, 2.9)	

Couple-level analysis: obesity: BMI ≥ 30; overweight: 30 > BMI ≥ 25.

^$^The sum of numbers among categories may not add up to 11254 due to missing data.

Testing for cross strata difference, compared to the referent stratum: ∗*P* < 0.05; ∗∗*P* < 0.01; ∗∗∗*P* < 0.001. (ref): the referent stratum.

^£^ *D*: difference in BMI = (husband's BMI − wife's BMI).

^†^Adjusted for individual level variables, including husband's and wife's ages, education years, race/ethnicity, physical activity, smoking, individual income, employment status, survey year, indicator of respondent, and missingness indicators.

**Table 3:** Association between obesity and one's own/the spouse's characteristics in the US (MEPS 2006–2008).

Explanatory variables	Husbands' obesity		Wives' obesity	
	OR	95% CI	OR	95% CI
Spouse's obesity status versus nonobesity	**2.52**	**(2.19, 2.89)**	**2.50**	**(2.18, 2.86)**
One's own age (per 5 years)	**0.92**	**(0.86, 0.98)**	1.06	(0.99, 1.13)
Spouse's age (per 5 years)	1.04	(0.97, 1.11)	0.97	(0.91, 1.04)
One's own HPA ≥3 times a week versus <3	**0.61**	**(0.53, 0.69)**	**0.56**	**(0.49, 0.63)**
Spouse's HPA ≥3 times a week versus <3	1.01	(0.90, 1.14)	1.13	(0.98, 1.30)
One's own current smoking versus nonsmoking status	**0.67**	**(0.57, 0.80)**	0.89	(0.72, 1.10)
Spouse's current smoking versus nonsmoking status	**1.30**	**(1.08, 1.56)**	1.19	(0.98, 1.45)
One's own race/ethnicity (versus NH white)				
NH black	**1.68**	**(1.09, 2.58)**	0.97	(0.59, 1.59)
Hispanic	0.97	(0.70, 1.34)	1.20	(0.90, 1.59)
Other	**0.51**	**(0.31, 0.85)**	**0.40**	**(0.23, 0.70)**
Spouse's race/ethnicity (versus NH white)				
NH black	0.75	(0.49, 1.16)	**2.09**	**(1.30, 3.36)**
Hispanic	0.83	(0.59, 1.15)	0.85	(0.63, 1.15)
Other	0.76	(0.49, 1.16)	0.88	(0.49, 1.58)
One's own education (per 5 year)	**0.86**	**(0.74, 0.99)**	0.92	(0.79, 1.07)
Spouse's education (per 5 year)	0.90	(0.79, 1.04)	**0.71**	**(0.61, 0.84)**
One's own individual income (versus ≥US$48,000)				
<US$10,000	0.94	(0.75, 1.18)	1.20	(0.96, 1.50)
US$10,000–24,999	1.02	(0.86, 1.21)	1.15	(0.95, 1.40)
US$25,000–47,999	1.01	(0.88, 1.17)	1.01	(0.83, 1.22)
Spouse's individual income (versus ≥US$48,000)				
<US$10,000	1.09	(0.90, 1.34)	1.17	(0.91, 1.49)
US$10,000–24,999	0.99	(0.83, 1.19)	**1.40**	**(1.18, 1.66)**
US$25,000–47,999	1.15	(0.97, 1.36)	**1.37**	**(1.17, 1.60)**
One's own employment status (versus employed)				
Never worked	0.68	(0.35, 1.34)	0.89	(0.68, 1.17)
Retired	0.95	(0.69, 1.32)	0.93	(0.61, 1.41)
Unemployed	1.10	(0.84, 1.44)	0.92	(0.75, 1.13)
Spouse's employment status (versus employed)				
Never worked	**0.69**	**(0.53, 0.89)**	1.06	(0.56, 2.00)
Retired	0.83	(0.56, 1.25)	0.73	(0.52, 1.04)
Unemployed	0.86	(0.72, 1.03)	**1.31**	**(1.01, 1.71)**

Individual-level analysis: obesity: BMI ≥ 30.

Each column shows the results of a logistic regression model; in addition to the variables presented on the table, survey year, indicator of respondent, and missingness indicators were adjusted.

**Table 4:** Association and similarity of habitual physical activity between husband and wife in the US: stratified by household-/couple-level characteristics.

	Crude % of having HPA			Kappa coefficients		Logistic regression		
	Husbands	Wives	Difference	*k*	95% CI	^†^OR	95% CI	
Whole sample	61.7%	58.2%	+3.5%	0.354	(0.353, 0.355)	4.5	(4.0, 5.0)	
Stratified by								
Couple's race/ethnicity								
Both NH white^(ref)^	64.2%	62.5%	+1.7%	0.333	(0.331, 0.335)	4.2	(3.7, 4.8)	
Both NH black	59.9%	51.5%	+8.4%	0.344	(0.333, 0.355)	4.6	(3.3, 6.3)	
Both Hispanic	50.3%	41.1%	+9.2%	0.397	(0.393, 0.400)	6.0	(4.8, 7.5)	∗
Both were other	55.7%	50.6%	+5.0%	0.366	(0.349, 0.383)	5.4	(3.6, 8.2)	
Interracial/ethnic	63.5%	58.9%	+4.6%	0.345	(0.332, 0.359)	5.2	(3.6, 7.5)	
Couple's mean age								
<30 y	65.8%	56.4%	+9.4%	0.362	(0.352, 0.372)	5.1	(3.7, 6.9)	
30–39 y	60.9%	57.1%	+3.9%	0.373	(0.370, 0.377)	4.9	(4.0, 6.0)	
40–54 y	60.8%	58.7%	+2.1%	0.348	(0.345, 0.351)	4.4	(3.7, 5.2)	
≥55 years	61.8%	59.7%	+2.1%	0.339	(0.335, 0.342)	4.2	(3.3, 5.3)	
Family size								
2 persons	63.9%	59.7%	+4.1%	0.357	(0.353, 0.361)	4.8	(3.9, 5.8)	
3-4 persons	61.7%	58.9%	+2.8%	0.329	(0.327, 0.331)	4.0	(3.5, 4.7)	∗
≥5 persons^(ref)^	57.6%	53.8%	+3.8%	0.407	(0.404, 0.410)	5.5	(4.4, 6.9)	
Employment condition								
Both unemployed	54.4%	49.5%	+4.9%	0.370	(0.359, 0.381)	5.2	(3.5, 7.7)	
Only husband unemployed	54.0%	56.9%	−2.9%	0.317	(0.298, 0.336)	4.5	(2.9, 7.2)	
Only wife unemployed	60.0%	57.0%	+3.0%	0.344	(0.340, 0.349)	4.5	(3.6, 5.6)	
Both employed^(ref)^	63.6%	59.7%	+4.0%	0.356	(0.354, 0.357)	4.6	(4.1, 5.3)	
Household income level								
<200% poverty line	53.7%	47.5%	+6.2%	0.404	(0.401, 0.408)	6.1	(4.9, 7.6)	∗
≥200% poverty line	63.5%	60.7%	+2.8%	0.335	(0.334, 0.337)	4.2	(3.7, 4.8)	
Receiving food stamp last year								
Ever	55.5%	50.2%	+5.3%	0.468	(0.454, 0.482)	7.9	(4.9, 12.6)	∗
No^(ref)^	61.9%	58.5%	+3.4%	0.349	(0.348, 0.351)	4.4	(4.0, 4.9)	

Couple-level analysis: HPA (habitual physical activity).

^†^Adjusted for individual level variables, including husband's and wife's ages, education years, race/ethnicity, weight status, smoking, individual income, employment status, survey year, indicator of respondent, and missingness indicators.

Testing for cross strata difference in OR, compared to the referent stratum: ∗*P* < 0.05. (ref): the referent stratum.

**Table 5:** Association between habitual physical activity (HPA) and one's own and the spouse's characteristics in the US (MEPS 2006–2008).

	Husbands' HPA			Wives' HPA		
	OR	95% CI		OR	95% CI	
Spouse's HPA (≥3 versus <3 times/week)	**4.54**	**(4.07, 5.06)**		**4.58**	**(4.11, 5.11)**	
One's own age (per 5 years)	**0.92**	**(0.87, 0.98)**		1.02	(0.96, 1.08)	
Spouse's age (per 5 years)	1.06	(0.998, 1.12)		1.00	(0.95, 1.06)	
One's own current smoking versus nonsmoking status	**0.73**	**(0.63, 0.85)**		**0.71**	**(0.61, 0.83)**	
Spouse's current smoking versus non-smoking status	**1.31**	**(1.10, 1.56)**		1.13	(0.97, 1.31)	
One's own race/ethnicity (versus NH white)						
NH black	1.34	(0.91, 1.98)		0.98	(0.65, 1.48)	
Hispanic	**0.78**	**(0.61, 0.99)**		0.79	(0.62, 1.001)	
Other	0.92	(0.65, 1.30)		0.72	(0.50, 1.02)	
Spouse's race/ethnicity (versus NH white)						
NH black	0.78	(0.52, 1.16)		0.77	(0.53, 1.14)	
Hispanic	1.03	(0.80, 1.32)		0.80	(0.63, 1.00)	
Other	0.84	(0.59, 1.19)		0.89	(0.61, 1.31)	
One's own education (per 5 year)	1.01	(0.90, 1.14)		**1.16**	**(1.02, 1.31)**	
Spouse's education (per 5 year)	1.11	(0.98, 1.25)		**1.19**	**(1.05, 1.34)**	
One's own individual income (versus ≥US$48,000)						
<US$10,000	1.11	(0.88, 1.41)		1.08	(0.88, 1.31)	
US$10,000–24,999	1.02	(0.86, 1.21)		0.94	(0.79, 1.10)	
US$25,000–47,999	**1.19**	**(1.03, 1.36)**		0.99	(0.84, 1.17)	
Spouse's individual income (versus ≥US$48,000)						
<US$10,000	0.96	(0.79, 1.17)		**0.69**	**(0.55, 0.87)**	
US$10,000–24,999	0.91	(0.77, 1.09)		0.87	(0.75, 1.01)	
US$25,000–47,999	1.00	(0.85, 1.17)		**0.83**	**(0.72, 0.96)**	
One's own employment status (versus employed)						
Never worked	0.78	(0.51, 1.20)		0.95	(0.77, 1.18)	
Retired	1.16	(0.87, 1.53)		1.08	(0.78, 1.51)	
Unemployed	**0.51**	**(0.40, 0.66)**		0.85	(0.72, 1.01)	
Spouse's employment status (versus employed)						
Never worked	0.87	(0.70, 1.08)		0.62	(0.37, 1.04)	
Retired	0.80	(0.57, 1.12)		**0.75**	**(0.57, 0.98)**	
Unemployed	1.05	(0.87, 1.26)		1.28	(0.996, 1.65)	

Individual-level analysis: HPA (habitual physical activity).

Each column shows the results of a logistic regression model; in addition to the variables presented on the table, survey year, indicator of respondent, and missingness indicators were adjusted.
